# Validating of a Nutrition and Food Security Screening Tool for Older Adults

**DOI:** 10.1093/geroni/igaf122.1658

**Published:** 2025-12-31

**Authors:** Lauri Wright, Jenna Pennella, Jenifer Ross, Paul Fuglestad

**Affiliations:** University of South Florida, Tampa, Florida, United States; University of North Florida, Jacksonville, Florida, United States; University of North Florida, Jacksonville, Florida, United States; University of North Florida, Jacksonville, Florida, United States

## Abstract

Food insecurity affects many older Americans due to factors such as financial constraints, transportation access, physical mobility, and bodily functioning. However, the most widely used measure for food insecurity, the USDA Household Food Security Survey Module (FSSM), primarily assesses financial security, overlooking other critical domains. This study aimed to develop and validate a screening tool that captures additional dimensions of food insecurity among older adults. We surveyed 72 older adults (ages 66–87). Each participant met with a registered dietitian nutritionist (RDN), who conducted a comprehensive nutritional assessment and administered both the USDA-FSSM and a newly developed scale based on the World Health Organization’s (WHO) International Classification of Functioning, Disability, and Health. Based on the nutritional assessment, the RDN categorized individuals as food secure, food insecure, or at risk for food insecurity. The FSSM explained only 34% of the variance in dietitian assessments, suggesting it does not fully capture food insecurity in this population. The newly developed scale demonstrated strong internal reliability (Cronbach’s alpha = .77). Factor analysis using Principal Axis Factoring with Oblimin rotation identified four key factors: mobility/physical limitations, access limitations, financial limitations, and bodily function issues. The overall scale score correlated strongly with the dietitian assessment (r = .69) and moderately with the FSSM (r = .51). These findings highlight that food insecurity in older adults extends beyond financial constraints. The newly developed screening tool effectively captures physical and functional challenges that impact food access and consumption, offering a more comprehensive assessment than the FSSM.

